# Gastrosplenic Fistula in the Setting of Undiagnosed Lymphoma: A Case Report

**DOI:** 10.5811/cpcem.34864

**Published:** 2025-02-26

**Authors:** Mackenzie Lecher, Brian Lecher, Lindsay Tjiattas-Saleski

**Affiliations:** * Edward Via College of Osteopathic Medicine, Department of Emergency Medicine, Spartanburg, South Carolina; † Faster Care Inc, Urgent Care, Sumter, South Carolina

**Keywords:** case report, fistula, splenomegaly, lymphoma, psoriasis

## Abstract

**Introduction:**

A gastrosplenic fistula (GSF) is a pathologic connection between the spleen and stomach that can lead to life-threatening complications. A GSF can arise spontaneously but is often secondary to a variety of etiologies. Most commonly, GSFs arise from gastric or splenic non-Hodgkin diffuse large B-cell lymphomas (DLBCL). Only 46 cases of GSFs have been published to date, and due to its rarity extensive literature review is insufficient for characterization of GSFs.

**Case Report:**

This case discusses a patient with intermittent abdominal pain and weight loss, which led to the diagnosis and treatment of a GSF and DLBCL. The patient later went into remission for his DLBCL but succumbed to respiratory failure from a secondary abdominal-pleural fistula formation. Gastrosplenic fistulas have the potential to cause fatal, massive, upper gastrointestinal hemorrhages, infections, other fistulas, or esophageal obstructions. A delay in diagnosis corresponds with a higher morbidity and mortality; thus, prompt detection and treatment are imperative. The management of GSFs is complex and requires a multidisciplinary approach to care.

**Conclusion:**

In this report we review GSFs in the emergency care setting with the goal of increasing awareness to facilitate their diagnosis.

## INTRODUCTION

While some anatomic fistulas are well known and even expected in certain disease states, gastrosplenic fistula (GSF) is a rare and potentially fatal entity. Among the various disease states that have been shown to cause a GSF, diffuse large B-cell lymphoma (DLBCL) is the most common.^1^ One potential explanation for this may be due to the lack of a desmoplastic reaction in lymphomas allowing rapid growth, gastric wall invasion, and tumor necrosis, a constellation not seen in adenocarcinomas.^2^ Other causes of GSF include chemotherapy-induced tumor lysis syndrome, diffuse histiocytic lymphoma, splenic abscess, peptic ulcer disease, Hodgkin lymphoma, gastric adenocarcinoma, extranodal natural killer/T-cell lymphoma, Crohn disease, sleeve gastrectomy, and trauma.^1^ Development of a GSF has been documented in less than 1% of gastric cancers.^2^ Diffuse large B-cell lymphoma can arise in any tissue, but the most common site of extranodal disease is found in the stomach.^3^

Formation of a GSF is often facilitated by splenic enlargement and its proximity to the gastric fundus.^4^ The GSF is often forged through a chronic process involving tissue necrosis secondary to lymphoma and infiltration into the gastric wall and splenic capsule.^1^ This direct communication between the stomach and spleen allows for the passage of gastric contents into the spleen, leaving the organ vulnerable to damage due to the acidity of gastric contents. Enlargement of the spleen due to inflammation and intraparenchymal air produced by tissue necrosis can irritate the diaphragm, potentially leading to pleural effusions, splenic perforation, abscesses, or splenopleural fistula formation.^5^

Clinically, GSFs have a wide spectrum of presentations from asymptomatic to hemorrhagic shock, which makes detection and diagnosis difficult. However, the most commonly reported symptom is abdominal pain.^6^ A worse prognosis is associated with an initial presentation of gastrointestinal (GI) bleeding, which often involves the splenic artery.^6^ Complications of GSF may include gastric perforation, infection, spleen destruction, abscess, pleural effusion, fistula formation with other organs, and more rapid metastasis.^6^

Abdominal computed tomography (CT) with contrast is the preferred imaging method for diagnosing GSF, but upper GI endoscopy can also provide direct visualization.^2^,^6^ Significant findings indicating a GSF include air in the spleen, splenomegaly, gastric ulceration, and communication between the spleen and stomach.^2^ Treatment varies depending on the presentation, extent of fistula formation, and organ damage. Currently, an approved treatment for hemodynamically stable, active bleeding is with splenic artery embolization performed by an interventional radiologist.^6^ Surgical resection including splenectomy and gastrectomy for treatment of GSF is generally considered first-line treatment, but due to the rarity of the condition there is no clear consensus on this.^2^

## CASE REPORT

A 59-year-old White male with a past medical history of hypertension, coronary artery disease, obesity, and psoriatic arthritis presented to an urgent care with intermittent left upper and lower quadrant abdominal pain, dark urine, fatigue, and nausea, which had gradually increased in severity over five weeks. Over the course of two months, the patient reported unintentional weight loss of 25 pounds but denied any melena, hematochezia, or tenesmus. Upon his arrival to the urgent care, he had a blood pressure of 145/88 millimeters of mercury, heart rate of 90 beats per minute, respiratory rate of 18 breaths per minute, temperature of 37 °Celsius, and 99% oxygen saturation on room air. The patient had moderate upper and lower abdominal tenderness on physical exam with normal bowel sounds and no palpable masses or organomegaly. Laboratory testing revealed a white blood cell count of 15.0 x10^3^ cells/microliter (μL) (reference range: 5–10 × 10^3^ cells/μL). All remaining lab values including hemoglobin, platelet count, basic metabolic panel, amylase, lipase, and coagulation studies were within normal limits.

**Image 3:**
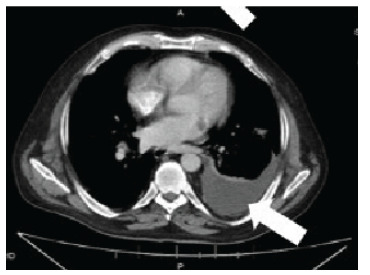
Computed tomography abdomen and pelvis showing a pleural effusion (arrow) of the left lung base with atelectasis.

A CT of the abdomen and pelvis with and without contrast was performed revealing a large heterogeneous-appearing spleen with gas appearing to extend from the body of the stomach into the splenic hilum (Image 3 shows a small, left-sided pleural effusion with atelectasis or infiltrate in the left lung base.

The diagnosis of a GSF was made, and the patient was admitted to the hospital where he underwent treatment for the next few weeks. During that time, an esophagogastroduodenoscopy was performed showing inflammation at the gastric fundus and a perforated ulcer with abscess formation and abnormal mucosa. A biopsy was obtained and tested positive for tumor markers and pathology consistent with DLBCL. During the surgery, a large 7.0 × 6.0-centimeter gastric tumor three inches inferior to the gastroesophageal junction was visualized. Perforation into the splenic capsule caused splenomegaly and tumor invasion to the diaphragm on the left, the pancreatic tail; the left lobe of the liver was also identified and treated during the procedure. The patient underwent a splenic embolization followed distal esophagectomy, total gastrectomy, omental pedicle flap, Roux-en-Y esophagojejunostomy, splenectomy, distal pancreatectomy, feeding jejunostomy tube placement, small bowel resection, and partial left hepatectomy. He tolerated the procedure well and was later treated by oncology for stage-III DLBCL with chemotherapy. Despite achieving remission, the patient died of complications three years later.

**CPC-EM Capsule**

What do we already know about this clinical entity?

*Gastrosplenic fistulas (GSF) are rare and often form in the presence of lymphoma. Initial symptoms can range from generalized abdominal pain to massive gastrointestinal hemorrhaging*.

What makes this presentation of disease reportable?

*This patient was previously undiagnosed, making it an unusual first presentation of diffuse large B-cell lymphoma*.

What is the major learning point?

*Due to the rarity and potentially fatal outcome of a GSF, it is imperative to expedite early detection and develop improved therapeutic strategies*.

How might this improve emergency medicine practice?

*Physicians should maintain a high index of suspicion for GSF, particularly in patients with diffuse large B-cell lymphoma*.

## DISCUSSION

This patient had a GSF secondary to DLBCL. Gastrosplenic fistulas have a wide range of presentations, which could be a potential cause of delayed treatment. Documented cases of GSFs have included the following presenting symptoms: abdominal pain, splenomegaly, constitutional symptoms, hematemesis, melena, gastric hematoma, hemorrhaging, splenic abscess, nausea, and weakness.^1^ Most documented cases of GSFs have been in males with an average age of 50 years and history of lymphoma.^1^

While GSF remains a rare occurrence, DLBCL is associated with numerous risk factors including the presence of DLBCL.^3^,^7^ Current data indicates that DLBCL occurs most frequently in males with a median age of 55 years who are White, and individuals with a body mass index greater than 30 kilograms per meter squared (kg/m^2^) (18.5–24.9 kg/m^2^), all of which put the patient in this case at increased risk.^3^ Recent studies have also shown a significant increase in incidence and mortality of DLBCL in individuals with an underlying history of autoimmune disease.^8^ While this association has often been connected to B-cell mediated diseases such as rheumatoid arthritis or systemic lupus erythematosus, one study from 2019 showed decreased survival rates due to non-Hodgkin lymphoma (NHL) in patients with psoriatic arthritis, a T-cell mediated disease.^8^ While it remains controversial, articles dating back to 1993 have provided evidence that methotrexate has been shown to cause lymphoproliferative diseases such as NHL that later went into remission with cessation of the drug; this phenomenon is a disease now termed methotrexate-associated lymphoproliferative disorder.^7^ There is some evidence of increased risk of lymphoma specifically in patients with psoriatic arthritis being treated with methotrexate.^9^ The patient in this case had been taking methotrexate to treat his psoriatic arthritis for five years, potentially placing him at increased risk for DLBCL.[Fig f2-cpcem-9-161]

Patients with a GSF treated with surgery generally have a good prognosis but often have a propensity for developing complications from surgery due to a weakened immune system.^1^,^10^ Surgical treatment of GSF varies but frequently involves both splenectomy and gastrectomy.^2^ Potential complications of a splenectomy include increased risk of infection with encapsulated organisms, bleeding, venous thromboembolism, and many cancers.^10^,^11^ Gastrectomy complications include anastomotic leakage, stenosis, dumping syndrome, abscesses, perforation, renal dysfunction, respiratory complications, and anemia.^12^ In a systematic review conducted in 2017, the most common cause of death from GSFs was gastric perforation followed by progression of lymphoma and pulmonary infection with multi-organ failure.^1^ Overall, when the initial presentation of the GSF is not in the setting of massive GI hemorrhaging, patients have an 82% survival rate.^6^

## CONCLUSION

Because they are rare, insufficient data exists regarding gastrosplenic fistulas; thus, it is important to document cases to expedite early detection and foster the development of improved therapeutic strategies. It is essential to keep a high level of suspicion for GSF in patients with DLBCL to prevent fatalities. Further studies should be conducted to increase awareness of GSFs and improve patient care.

## Figures and Tables

**Image 1:**
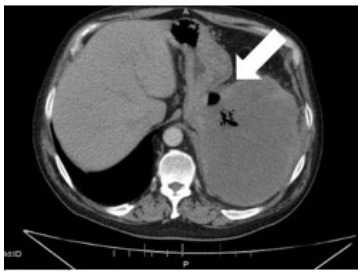
Computed tomography abdomen and pelvis axial view displaying the presence of gas (arrow) within the spleen.

**Image 2 f2-cpcem-9-161:**
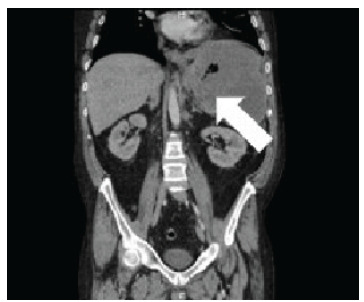
Computed tomography abdomen and pelvis coronal image showing the presence of gas (arrow) within the spleen.
